# Informal Treatment Practices in Ornamental Aquaria: An Overlooked Interface Between Aquatic Animal Health, Antimicrobial Stewardship, and One Health

**DOI:** 10.3390/ani16132056

**Published:** 2026-07-03

**Authors:** Marco Dettori

**Affiliations:** Department of Medicine, Surgery and Pharmacy, University of Sassari, 07100 Sassari, Italy; madettori@uniss.it

**Keywords:** ornamental aquaria, ornamental fish, aquatic animal health, animal welfare, captive animal management, antimicrobial stewardship, One Health, informal treatment practices, aquarium microbiome

## Abstract

Ornamental aquarium keeping collectively involves millions of freshwater, marine, and reef systems in which fish, corals, invertebrates, biofilters, microbial communities, and human husbandry practices are closely interconnected. In these domestic aquatic animal systems, preventive and curative treatments may include antimicrobials, antiparasitics, antiseptics, oxidizing agents, copper-based products, dips, and commercial formulations targeting microbial proliferations or visible system deterioration. Many interventions occur without veterinary diagnosis, microbiological confirmation, standardized dosing, active-ingredient transparency, or post-treatment monitoring. This raises concerns for aquatic animal health and welfare, as whole-system treatments may affect not only the intended pathogen or pest but also non-target organisms, biofilter communities, animal-associated microbiota, and water quality stability. Digital communities and online platforms can rapidly circulate empirical treatment protocols, although they may also provide opportunities for stewardship education and improved husbandry guidance. Current evidence does not support interpreting ornamental aquaria as major independent drivers of antimicrobial resistance. The more defensible concern is stewardship: biologically active compounds may be used repeatedly and empirically in animal systems without diagnosis, professional guidance, or systematic monitoring. This Perspective argues that ornamental aquaria should be recognized as an overlooked interface between aquatic animal health, welfare, antimicrobial stewardship, and One Health. It proposes a research and communication agenda focused on treatment transparency, diagnosis, prevention, biofilter protection, and responsible care practices.

## Simple Summary

Ornamental aquaria are widely kept domestic aquatic systems that contain fish, corals, invertebrates, biofilters, and complex microbial communities. When diseases, visible blooms, parasites, or poor water quality occur, hobbyists often use antimicrobial, antiparasitic, antiseptic, or other biologically active products, frequently without veterinary diagnosis or microbiological confirmation. These treatments may affect not only the intended pathogen or pest, but also animal welfare, non-target organisms, biofilter stability, and the microbial balance of the aquarium. Online communities can spread empirical treatment protocols, but they may also offer opportunities for better education and stewardship. This Perspective argues that ornamental aquaria deserve greater attention as an overlooked interface between aquatic animal health, welfare, antimicrobial stewardship, and One Health.

## 1. Introduction

Millions of ornamental aquaria are maintained worldwide; marine aquaria alone were estimated to involve about two million hobbyists globally [1]. These systems include freshwater, marine, and reef aquaria maintained in private households, retail facilities, breeding centres, public aquaria, and online supply chains [2,3,4,5,6]. Although commonly treated as recreational or commercial objects, aquaria are managed aquatic animal systems in which fish, corals, invertebrates, water chemistry, biofilms, microbial succession, and human intervention are tightly connected.

Informal treatment practices in ornamental aquaria include interventions selected and applied without formal veterinary diagnosis or supervision, often on the basis of product labels, retailer advice, online forums, social media, or accumulated hobbyist experience. Their One Health relevance derives from the intersection of aquatic animal health and welfare, antimicrobial use, microbial ecology, household practices, and the potential disposal of treated water or biological material into domestic waste streams.

From an animal-health perspective, these systems are not only decorative environments but managed habitats for living aquatic animals and associated microbial communities. Treatment decisions may therefore have welfare implications, particularly when disease, stress, poor water quality, or misdiagnosis leads to repeated treatment of the aquarium as a whole. In this context, treatment applied directly to the aquarium water may expose target animals, non-target organisms, biofilms, microbial communities, and filtration processes simultaneously [5,6,7].

The biological stability of an aquarium depends on structured microbial processes. Nitrifying communities in biofilters transform toxic ammonia into less toxic oxidized nitrogen compounds and are shaped by substrate, oxygen availability, organic loading, water circulation, and system maturation [7,8,9]. Interventions directed at fish disease, quarantine, water quality, biofilm control, visible blooms, nuisance organisms, or visual cleanliness may therefore affect not only the intended target, but also the ecological architecture of the system.

Antimicrobial exposure in ornamental aquaria remains poorly framed. Antibiotics and other biologically active products may be used for bacterial disease treatment, quarantine, prophylaxis, transport-associated stress, retail losses, or non-specific attempts to correct deteriorating tank conditions [10,11,12,13,14]. Evidence from the ornamental fish sector already indicates antimicrobial use, antimicrobial-resistant bacteria, and zoonotic bacteria across production, importation, retail, and household-related contexts [10,11,12,14,15,16].

Yet ornamental aquaria remain peripheral in antimicrobial resistance debates, which still focus mainly on human medicine, livestock, food-producing aquaculture, wastewater, agriculture, pharmaceutical contamination, and environmental reservoirs [17,18]. This Perspective argues that ornamental aquaria should be considered neglected domestic aquatic animal systems where animal health, welfare, consumer products, environmental microbiology, digital communication, and informal treatment practices converge.

## 2. Ornamental Aquaria as Domestic Aquatic Animal and Microbial Systems

Ornamental aquaria are not sterile water containers. They are domestic microbial and animal-care systems maintained through filtration, animal feeding, water exchange, lighting, circulation, substrate management, and chemical correction [6,7,8,9,19]. Their apparent stability depends on microbial and ecological processes that are largely invisible to the user [7,8,9].

The biofilter is the main microbial infrastructure of the aquarium. Nitrifying communities oxidize ammonia and nitrite, while heterotrophic bacteria degrade organic matter and contribute to nutrient turnover [7,8,9]. These communities develop on filter media, substrates, glass, rocks, sediments, pipes, pumps, and animal-associated surfaces [7,8,9]. Their composition is shaped by oxygen availability, organic load, flow, temperature, pH, salinity, and system age [7,9].

Disturbance is common. Overfeeding, animal introduction, transport stress, mortality events, incomplete maturation, excessive cleaning, inadequate flow, unstable nutrient inputs, and changes in bioload can alter microbial succession and water chemistry [6,9,13,14]. In newly established systems, transient blooms and unstable nitrogen dynamics are frequent because biofilter communities are still developing [9]. In mature systems, similar instability may follow changes in husbandry, stocking density, feeding, or maintenance practices [13,14].

These disturbances are usually recognized through visible or clinical signs. Fish may develop fin erosion, skin lesions, ulcers, abnormal mucus production, respiratory distress, behavioural changes, fungal-like growths, or external parasitic disease [13,14,15,20]. White spot disease, commonly associated with *Ichthyophthirius multifiliis* in freshwater systems and *Cryptocaryon irritans* in marine systems, is one familiar example because it produces visible lesions and often triggers rapid treatment decisions [13,20,21,22]. Other recurrent problems include bacterial infections, ectoparasites, intestinal worms, dinoflagellate blooms, diatom films, filamentous algae, cyanobacterial mats, nuisance anemones such as *Aiptasia*, flatworms, hydroids, and other unwanted organisms in reef systems [13,15,23,24,25,26,27].

The diagnosis is usually visual. Hobbyists may classify a problem based on colour, texture, location, animal behaviour, lesion morphology, water appearance, or the presence of bubbles, films, spots, worms, or visible pests [13,14,19,22]. This distinction is often uncertain. Similar signs may derive from different causes, and several problems may coexist in the same system [13,15]. A tank with deteriorating water quality, for example, may simultaneously show fish stress, opportunistic infection, algal growth, biofilm accumulation, and parasite susceptibility [13,14,15].

This diagnostic uncertainty matters because treatment acts on the whole system. Antibacterial, antifungal, antiparasitic, disinfectant, algaecidal, anti-protozoal, deworming, and broad-spectrum remedy products are not applied to isolated pathogens [10,11,12,13,14]. They enter aquaria containing nitrifiers, heterotrophs, biofilms, phototrophic microorganisms, animal microbiota, sediments, invertebrates, plants, or corals, as well as dissolved organic matter [7,8,9,23]. Their effects may therefore extend beyond the visible target, especially when products are used repeatedly, preventively, or in response to non-specific deterioration [10,11,12,13,14].

Repeated or poorly targeted treatment introduces biological pressure into dense, biofilm-rich aquatic animal systems [10,11,12,13,14,17]. From an animal perspective, the central concern is not only antimicrobial resistance, but also the welfare of affected animals, the stability of biofilters, the exposure of non-target organisms, and the potential normalization of treatment before diagnosis. Table 1 summarizes recurrent aquarium problems through which these concerns may emerge. 

## 3. Informal Treatment Practices and Animal Welfare Concerns

Aquarium treatment practices often develop outside formal veterinary or microbiological frameworks. Hobbyists commonly rely on product labels, retailer advice, online forums, social media groups, and accumulated community experience to select interventions for fish disease, quarantine, parasites, visible blooms, nuisance organisms, or deteriorating water quality [13,14,19,28,29,30]. This produces a practical but weakly standardized treatment culture in which empirical correction often precedes diagnosis [13,14,20].

The products used in this context are heterogeneous. They include antibacterial, antifungal, antiparasitic, anti-protozoal, deworming, disinfectant, algaecidal, anti-cyanobacterial, and broad-spectrum general-cure formulations [13,14,20,22]. Some are used for clinically apparent disease, whereas others are applied preventively during quarantine, after transport, after animal introduction, or in response to non-specific signs of system deterioration [10,11,12,13,15,20]. In many cases, the intervention is directed at the aquarium as a whole rather than at an identified pathogen [13,14].

Product transparency remains a critical limitation. Hobbyists may apply biologically active substances without knowing their precise composition, concentration, spectrum of activity, pharmacological class, or ecological effects [10,12,13,14]. Product communication often emphasizes rapid visual improvement rather than diagnosis, microbial selectivity, resistance risk, welfare implications, or system-level consequences [13,19,30]. In biofilm-rich aquaria, this means that nitrifying bacteria, heterotrophs, phototrophic microorganisms, animal microbiota, sediments, plants, corals, and invertebrates may all be exposed to the same treatment [7,8,9,23].

These practices are poorly aligned with antimicrobial stewardship principles. In clinical and veterinary settings, antimicrobial use is expected to rely on indication, diagnosis, appropriate compound selection, dosage, duration, and resistance prevention [17,18]. In ornamental aquaria, antimicrobial or antimicrobial-like products may instead be introduced without microbiological testing, susceptibility assessment, standardized protocols, post-treatment monitoring, or evaluation of ecological and welfare consequences [10,11,12,13,14,18]. The gap is not only regulatory. It is conceptual: the aquarium is often treated as a visual problem to be corrected, while it functions as an animal system and microbial ecosystem under selective pressure [7,8,9,17].

Recurrence reinforces this pattern. Visible improvement after treatment may be interpreted as resolution, even when poor water quality, excessive organic load, unstable nutrients, inadequate flow, immature filtration, overstocking, or repeated animal introduction continue to favour disease, blooms, or opportunistic proliferation [7,9,13,14,24,25,26,27]. Temporary suppression can therefore normalize repeated treatment cycles, particularly when products are accessible, community-endorsed, and perceived as low-risk [13,28,29,30].

Informal treatment practices in ornamental aquaria represent a relevant but underexamined source of biological pressure within domestic aquatic animal systems, with potential implications for animal health, welfare, biofilter stability, and antimicrobial stewardship. Their importance lies in the convergence of dense microbial communities, recurrent empirical treatment, limited product transparency, online-mediated decision-making, and absence of stewardship mechanisms [12,13,14,17,18].

## 4. Digital Communities and Circulation of Treatment Protocols

The aquarium hobby is strongly shaped by digital communication. Online forums, blogs, video platforms, social media groups, and specialized websites are major sources of technical information for hobbyists, including recommendations on filtration, stocking, quarantine, feeding, nutrient control, pest management, disease treatment, microbial blooms, and chemical interventions [19,28,29,30]. Digital communities therefore function as practical knowledge systems in which treatment decisions are discussed, compared, modified, and redistributed.

This circulation is not limited to conventional antimicrobials. It also includes general-cure formulations, antiparasitic protocols, copper- or formalin-based treatments, disinfectants, anti-algal and anti-cyanobacterial products, herbal extracts, essential oils, garlic-enriched feeds, and other products perceived as natural or low-risk [13,20,22]. Garlic-derived products illustrate this grey area, as *Allium sativum* has been investigated in aquaculture as a feed additive, immunostimulant, and health-promoting supplement in several fish species [31]. Tea tree oil-derived products occupy a similar position: they are marketed or discussed as natural remedies, while the essential oil literature reports antimicrobial and antiparasitic biological activity [32,33].

Digital protocols are persuasive because they are operational. Users facing visible deterioration can rapidly find dosing schedules, product combinations, before-and-after images, anecdotal success reports, and advice on repeated treatment cycles. These elements may create a sense of collective validation even when the diagnosis, active ingredient, dose–response relationship, welfare consequence, or ecological effect remains uncertain [28,29,30]. The issue is not peer support itself, but its conversion into quasi-therapeutic protocols without diagnostic control.

Online communities can also promote good husbandry. Experienced hobbyists often recommend quarantine, improved water quality, nutrient stabilization, reduced organic load, better flow, manual removal, and avoidance of unnecessary chemical intervention [13,19]. These communities should therefore not be viewed only as sources of risk. Many aquarium hobbyists are strongly motivated by animal care, ecological balance, and daily observation of their systems. This engagement may make them particularly receptive to welfare-oriented husbandry, biofilter protection, responsible treatment use, and stewardship education. In this sense, hobbyist communities may represent practical channels for translating One Health and stewardship principles into everyday animal-care practices. This is particularly relevant in digital environments, where health-related information, perceptions, emotions, and behaviours can be rapidly amplified and shaped by web-based communication [34,35,36].

These dynamics are relevant to antimicrobial stewardship because digital communities can accelerate treatment practices beyond professional oversight. Aquarium communities operate as informal educational and regulatory environments: they define what counts as a problem, which products are considered acceptable, how protocols are adapted, and when repeated use becomes normal [10,11,12,13,14,19]. In this setting, antimicrobial exposure may be shaped as much by communication pathways as by product availability.

A One Health approach to ornamental aquaria should therefore include digital communication as part of the stewardship pathway. Treatment practices emerge through the interaction of consumer products, animal health concerns, microbial ecosystems, household decision-making, and online-mediated expertise. Engagement among environmental microbiologists, aquatic veterinarians, public health researchers, industry, and hobbyist communities may help shift informal protocols away from unnecessary biological pressure and toward diagnosis, prevention, ecological stabilization, and responsible animal care [17,18].

## 5. One Health and Antimicrobial Stewardship Implications

From an aquatic animal health perspective, ornamental aquaria occupy an unusual position. They are companion-animal systems, microbial ecosystems, and consumer-managed treatment environments. This makes them relevant to One Health, not because they are proven major drivers of antimicrobial resistance, but because they illustrate how animal care, informal pharmaceutical use, microbial ecology, and household practices can intersect outside formal stewardship systems.

Repeated, empirical, or poorly targeted antimicrobial exposure may create selective pressure within aquarium microbial communities and favour the persistence or selection of resistant organisms. This concern should remain proportionate: current evidence does not identify domestic ornamental aquaria as major independent drivers of antimicrobial resistance, but it supports greater attention to their stewardship and microbiological implications.

Antimicrobial resistance is recognized as a systemic problem involving human health, animal health, environmental contamination, microbial ecology, and social behaviour [17,18]. Ornamental aquaria remain marginal within this framework, although they combine companion animal care, biofilm-rich microbial communities, consumer-directed treatment practices, and possible interaction with domestic wastewater systems [10,12,14].

Their relevance lies in a specific convergence of factors: dense microbial communities, repeated husbandry interventions, empirical product use, limited diagnostic control, and possible disposal of treated water, filter material, sediments, biofilms, dead organisms, and treatment residues into household wastewater or solid waste streams [7,8,9,13,14,20]. These exposures occur in small systems, but under conditions where selective pressure, biofilm persistence, microbial succession, and repeated treatment cycles are biologically plausible concerns [17,18].

Existing studies provide concrete evidence of this interface. Research conducted in Hong Kong has documented antibiotic use in ornamental fish and antimicrobial resistance among associated zoonotic bacteria, while investigations of retail tanks have shown that prophylactic antibiotic exposure can alter microbial communities and favour pathogen selection in carriage water [10,11,12]. Resistant bacterial strains have also been isolated from imported ornamental fish in Italy, and systematic reviews have identified antimicrobial use, zoonotic bacteria, and antimicrobial resistance across production, importation, retail, and household-related settings [14,15,16]. These findings do not demonstrate that domestic aquaria are major contributors to the global antimicrobial-resistance burden, but they confirm that resistant organisms and selective pressures can occur within the ornamental fish pathway.

A proportionate One Health agenda should prioritize treatment transparency, reduced unnecessary antimicrobial exposure, improved communication on diagnosis and prevention, and targeted research on the microbial and welfare effects of commonly used aquarium products [13,17,18]. Ornamental aquaria are small but globally distributed systems in which aquatic animal health, microbial ecology, consumer behaviour, and antimicrobial stewardship intersect.

## 6. Conclusions

Ornamental aquaria are globally distributed domestic aquatic animal systems in which fish, corals, invertebrates, biofilters, consumer products, online-mediated expertise, and informal treatment practices intersect. Although their direct contribution to antimicrobial resistance remains uncertain, repeated empirical use of biologically active products in biofilm-rich animal systems deserves greater scientific and stewardship attention.

Priority actions should include clearer disclosure of active ingredients and dosing information; stronger differentiation between therapeutic products and products intended to correct ecological imbalance; reduced empirical and preventive antimicrobial use without diagnosis; improved access to aquatic-animal-health and veterinary guidance; and clearer communication on quarantine, prevention, water quality, and biofilter protection. Research should quantify antimicrobial exposure and resistance patterns in domestic aquaria; evaluate effects on animal welfare, nitrifying communities, biofilms, opportunistic pathogens and resistance genes; and assess the consequences of disposing of treated water and filter material through household waste systems. Engagement with retailers, industry, aquatic veterinarians, microbiologists, and digital hobbyist communities should be used to translate these priorities into practical stewardship guidance.

Recognizing ornamental aquaria within One Health does not require overstating their impact. It requires acknowledging a neglected interface in which aquatic animal care, microbial ecology, consumer behaviour, and antimicrobial stewardship converge outside conventional veterinary and public health systems.

## Figures and Tables

**Table 1:** Recurrent ornamental aquarium problems as entry points for informal treatment practices, aquatic animal welfare concerns, and stewardship gaps.

Recurrent Problem	Typical Hobbyist Recognition	Common Intervention Category	Aquatic Animal Health, Welfare, and Stewardship Concern
Bacterial disease, fin rot, ulcers	Lesions, fin erosion, abnormal mucus, lethargy	Antibacterial products, broad-spectrum remedies	Delayed diagnosis, empirical antibacterial use, lesion progression, stress, and no susceptibility testing
White spot disease/ich	Visible white spots, scratching, respiratory distress	Anti-protozoal products, copper- or formalin-based treatments, tank-wide medication	Respiratory distress, whole-system medication, toxicity in sensitive species, misdiagnosis, and non-target stress
Fungal-like growths	Cotton-like lesions	Antifungal products, general-cure formulations	Possible confusion with bacterial infection or tissue lesions, delaying appropriate care
Internal worms/ectoparasites	Weight loss, abnormal faeces, visible worms, scratching	Dewormers, antiparasitic products	Repeated empirical treatment, non-target effects, and stress in weakened animals
Dinoflagellate/diatom blooms	Brown films, bubbles, dust-like coatings	Algaecides, blackout, chemical correctors	Misclassification of ecological imbalance; possible effects on biofilter, corals, invertebrates, and water stability
Cyanobacterial mats/red slime	Red, blue-green, brown, or mucilaginous mats	Anti-cyanobacterial products, erythromycin or macrolide-like treatments	Antimicrobial or antimicrobial-like use for ecological imbalance; possible effects on biofilter, microbiota, corals, and invertebrates
Reef pests, e.g., Aiptasia, flatworms, hydroids	Visible unwanted organisms	Pest-control products, dips, biological control, chemical spot treatments	Local or whole-system interventions may affect non-target invertebrates, corals, microbial balance, and water chemistry

## Data Availability

No new data were generated or analyzed for this Perspective. All sources discussed in this article are cited in the reference list.

## References

[1] UNEP-WCMC and Marine Aquarium Council (2003). From Ocean to Aquarium: The Global Trade in Marine Ornamental Species.

[2] Biondo M.V., Aguayo F., Calado R. (2024). An updated review of the marine ornamental fish trade in the European Union. Animals.

[3] Biondo M.V., Burki R.P. (2020). A systematic review of the ornamental fish trade with emphasis on coral reef fishes—An impossible task. Animals.

[4] Leal M.C., Vaz M.C.M., Puga J., Rocha R.J.M., Brown C., Rosa R., Calado R. (2016). Marine ornamental fish imports in the European Union: An economic perspective. Fish Fish..

[5] Rhyne A.L., Tlusty M.F., Schofield P.J., Kaufman L., Morris J.A., Bruckner A.W. (2012). Revealing the appetite of the marine aquarium fish trade: The volume and biodiversity of fish imported into the United States. PLoS ONE.

[6] Tlusty M. (2002). The benefits and risks of aquacultural production for the aquarium trade. Aquaculture.

[7] Godzieba M., Hliwa P., Ciesielski S. (2025). Network of nitrifying bacteria in aquarium biofilters: An unfaltering cooperation between comammox Nitrospira and ammonia-oxidizing archaea. Water.

[8] McKnight M.M., Neufeld J.D. (2024). Comammox Nitrospira among dominant ammonia oxidizers within aquarium biofilter microbial communities. Appl. Environ. Microbiol..

[9] McKnight M.M., Szabolcs N., Graham A., Neufeld J.D. (2025). Microbial community succession of home aquarium biofilters associated with early establishment of comammox Nitrospira. ISME Commun..

[10] Au-Yeung C., Lam K.-L., Chan K.-W., Mo W.-Y. (2022). Uses of antibiotics in ornamental fish in Hong Kong and the antibiotic resistance in the associated zoonotic pathogens. J. Xenobiotics.

[11] Au-Yeung C., Lam K.-L., Choi M.-H., Chan K.-W., Cheung Y.-S., Tsui Y.-L., Mo W.-Y. (2024). Impact of prophylactic antibiotic use in ornamental fish tanks on microbial communities and pathogen selection in carriage water in Hong Kong retail shops. Microorganisms.

[12] Au-Yeung C., Tsui Y.-L., Choi M.-H., Chan K.-W., Wong S.-N., Ling Y.-K., Lam C.-M., Lam K.-L., Mo W.-Y. (2025). Antibiotic Abuse in Ornamental Fish: An Overlooked Reservoir for Antibiotic Resistance. Microorganisms.

[13] Larcombe E., Alexander M.E., Snellgrove D., Henriquez F.L., Sloman K.A. (2025). Current disease treatments for the ornamental pet fish trade and their associated problems. Rev. Aquac..

[14] Weir M., Rajić A., Dutil L., Cernicchiaro N., Uhland F.C., Mercier B., Tuševljak N. (2012). Zoonotic bacteria, antimicrobial use and antimicrobial resistance in ornamental fish: A systematic review of the existing research and survey of aquaculture-allied professionals. Epidemiol. Infect..

[15] Duman M., Satıcıoğlu İ.B., Janda J.M. (2024). A review of the industrial importance, common bacterial diseases, and zoonotic risks of freshwater aquarium fish. Vector-Borne Zoonotic Dis..

[16] Sicuro B., Pastorino P., Barbero R., Barisone S., Dellerba D., Menconi V., Righetti M., De Vita V., Prearo M. (2020). Prevalence and antibiotic sensitivity of bacteria isolated from imported ornamental fish in Italy: A translocation of resistant strains?. Prev. Vet. Med..

[17] Arnold K.E., Laing G., McMahon B.J., Fanning S., Stekel D.J., Pahl O., Coyne L., Latham S.M., McIntyre K.M. (2024). The need for One Health systems-thinking approaches to understand multiscale dissemination of antimicrobial resistance. Lancet Planet. Health.

[18] United Nations Environment Programme (2023). Bracing for Superbugs: Strengthening Environmental Action in the One Health Response to Antimicrobial Resistance.

[19] Marchio E.A. (2018). The art of aquarium keeping communicates science and conservation. Front. Commun..

[20] Cardoso P.H.M., Moreno A.M., Moreno L.Z., de Oliveira C.H., de Assis Baroni F., de Lucca Maganha S.R., de Souza R.L.M., Balian S.C. (2019). Infectious diseases in aquarium ornamental pet fish: Prevention and control measures. Braz. J. Vet. Res. Anim. Sci..

[21] Dickerson H.W., Woo P.T.K. (2006). *Ichthyophthirius multifiliis* and *Cryptocaryon irritans* (Phylum Ciliophora). Fish Diseases and Disorders, Volume 1: Protozoan and Metazoan Infections.

[22] Petty B.D., Francis-Floyd R., Yanong R.P.E. (2024). Management of aquarium fish. MSD Veterinary Manual.

[23] Bourne D.G., Morrow K.M., Webster N.S. (2016). Insights into the coral microbiome: Underpinning the health and resilience of reef ecosystems. Annu. Rev. Microbiol..

[24] Bozan M., Berreth H., Lindberg P., Bühler K. (2025). Cyanobacterial biofilms: From natural systems to applications. Trends Biotechnol..

[25] Feng L., Wang Y., Hou X., Qin B., Kutser T., Qu F., Chen N., Paerl H.W., Zheng C. (2024). Harmful algal blooms in inland waters. Nat. Rev. Earth Environ..

[26] Huisman J., Codd G.A., Paerl H.W., Ibelings B.W., Verspagen J.M.H., Visser P.M. (2018). Cyanobacterial blooms. Nat. Rev. Microbiol..

[27] Sukenik A., Kaplan A. (2021). Cyanobacterial harmful algal blooms in aquatic ecosystems: A comprehensive outlook on current and emerging mitigation and control approaches. Microorganisms.

[28] Chou W.Y.S., Hunt Y.M., Beckjord E.B., Moser R.P., Hesse B.W. (2009). Social media use in the United States: Implications for health communication. J. Med. Internet Res..

[29] Johansson V., Islind A.S., Lindroth T., Angenete E., Gellerstedt M. (2021). Online communities as a driver for patient empowerment: Systematic review. J. Med. Internet Res..

[30] Nan X., Wang Y., Thier K. (2022). Why do people believe health misinformation and who is at risk? A systematic review of individual differences in susceptibility to health misinformation. Soc. Sci. Med..

[31] Lee J.-Y., Gao Y. (2012). Review of the application of garlic, Allium sativum, in aquaculture. J. World Aquac. Soc..

[32] Carson C.F., Hammer K.A., Riley T.V. (2006). Melaleuca alternifolia (tea tree) oil: A review of antimicrobial and other medicinal properties. Clin. Microbiol. Rev..

[33] Lam N.S.K., Long X., Li X., Saad S., Lim F., Doery J.C.G., Griffin R.C. (2020). Melaleuca alternifolia (tea tree) oil and its monoterpene constituents in treating protozoan and metazoan parasitic infections. Biomed. Pharmacother..

[34] Dettori M., Arru B., Azara A., Piana A., Mariotti G., Camerada M.V., Stefanelli P., Rezza G., Castiglia P. (2018). In the Digital Era, Is Community Outrage a Feasible Proxy Indicator of Emotional Epidemiology? The Case of Meningococcal Disease in Sardinia, Italy. Int. J. Environ. Res. Public Health.

[35] Deiana G., Arghittu A., Dettori M., Castiglia P. (2024). One World, One Health: Zoonotic Diseases, Parasitic Diseases, and Infectious Diseases. Healthcare.

[36] Arghittu A., Deiana G., Dettori M., Dempsey E., Masia M.D., Palmieri A., Spano A.L., Azara A., Castiglia P. (2021). Web-based analysis on the role of Digital Media in Health Communication: The experience of VaccinarSinSardegna Website. Acta Biomed..

